# Discriminating Survival Outcomes in Patients with Glioblastoma Using a Simulation-Based, Patient-Specific Response Metric

**DOI:** 10.1371/journal.pone.0051951

**Published:** 2013-01-23

**Authors:** Maxwell Lewis Neal, Andrew D. Trister, Tyler Cloke, Rita Sodt, Sunyoung Ahn, Anne L. Baldock, Carly A. Bridge, Albert Lai, Timothy F. Cloughesy, Maciej M. Mrugala, Jason K. Rockhill, Russell C. Rockne, Kristin R. Swanson

**Affiliations:** 1 Department of Pathology, University of Washington, Seattle, Washington, United States of America; 2 Department of Radiation Oncology, University of Washington, Seattle, Washington, United States of America; 3 Department of Neurological Surgery, Northwestern University, Chicago, Illinois, United States of America; 4 Northwestern Brain Tumor Institute, Chicago, Illinois, United States of America; 5 Department of Neurology, University of California Los Angeles, Los Angeles, California, United States of America; 6 Department of Neurology, University of Washington, Seattle, Washington, United States of America; 7 Department of Applied Mathematics, University of Washington, Seattle, Washington, United States of America; 8 Nancy and Buster Alvord Brain Tumor Center, University of Washington, Seattle, Washington, United States of America; University of California-San Francisco, United States of America

## Abstract

Accurate clinical assessment of a patient's response to treatment for glioblastoma multiforme (GBM), the most malignant type of primary brain tumor, is undermined by the wide patient-to-patient variability in GBM dynamics and responsiveness to therapy. Using computational models that account for the unique geometry and kinetics of individual patients' tumors, we developed a method for assessing treatment response that discriminates progression-free and overall survival following therapy for GBM. Applying these models as untreated virtual controls, we generate a patient-specific “Days Gained” response metric that estimates the number of days a therapy delayed imageable tumor progression. We assessed treatment response in terms of Days Gained scores for 33 patients at the time of their first MRI scan following first-line radiation therapy. Based on Kaplan-Meier analyses, patients with Days Gained scores of 100 or more had improved progression-free survival, and patients with scores of 117 or more had improved overall survival. Our results demonstrate that the Days Gained response metric calculated at the routinely acquired first post-radiation treatment time point provides prognostic information regarding progression and survival outcomes. Applied prospectively, our model-based approach has the potential to improve GBM treatment by accounting for patient-to-patient heterogeneity in GBM dynamics and responses to therapy.

## Introduction

Glioblastoma multiforme (GBM) is an aggressive, highly invasive brain tumor with dynamic and spatial features that vary widely between patients [1], [2], [3], [4]. To make informed decisions about a patient's course of treatment, clinicians must be able to accurately assess patient response to surgical resection, chemotherapy and radiotherapy; however, this assessment is challenging because of the variability in proliferative and invasive dynamics of the disease. To provide estimates of a patient's progression-free survival (PFS) and overall survival (OS) following therapy, we developed a new, model-based response metric that accounts for the unique dynamics of each patient's tumor. Our metric, called “Days Gained,” differs from established response metrics such as the Macdonald [5], Response Evaluation Criteria in Solid Tumors (RECIST) [6], and Response Assessment in Neuro-Oncology (RANO) criteria [7] in that it accounts for 1) the presence of tumor cells peripheral to the abnormalities observed on clinical imaging; 2) irregularities in tumor shape; and 3) patient-specific rates of tumor growth. The first feature allows us to gauge the extent of a tumor's invasive characteristics beyond the threshold of MRI detection. The second incorporates the full 3D geometry of the tumor into the measure of response. The third provides an estimate of how large the tumor would have been at a certain time if left untreated. By incorporating the individually measured rate of tumor growth into our response metric, we reduce type II errors in identifying patients that respond to therapy. For example, a fast growing tumor that has a robust, pathological response to therapy might be classified as clinically progressing if one only considers the change in tumor burden between the pre-treatment and post-treatment MRIs (as specified by established response criteria). Instead, our method allows a comparison between the patient's tumor burden at a post-treatment time point and the *expected* tumor burden predicted by our model - a more complete and accurate assessment of a therapy's effectiveness.

The strength of existing response metrics, such as RANO, lies in their utility to confirm progression or response at the time of clinical follow-up in a standardized way. These metrics are also widely used to identify patients for clinical trials by retrospectively confirming tumor progression. In contrast, we created the Days Gained metric in the hopes of providing *prognostic* information to help clinicians optimize a patient's course of treatment [8]. In the longer term, we are interested in developing novel clinical tools that can quantify the effectiveness of a given therapy on a patient-by-patient basis, can provide predictive information to assist post-therapy decision-making, and can identify therapy-resistant patients as candidates for clinical trials immediately following treatment. The current study, which demonstrates the discriminating power of our Days Gained metric, represents a key step towards developing the predictive, patient-specific modeling tools that we envision.

**Figure 1:**
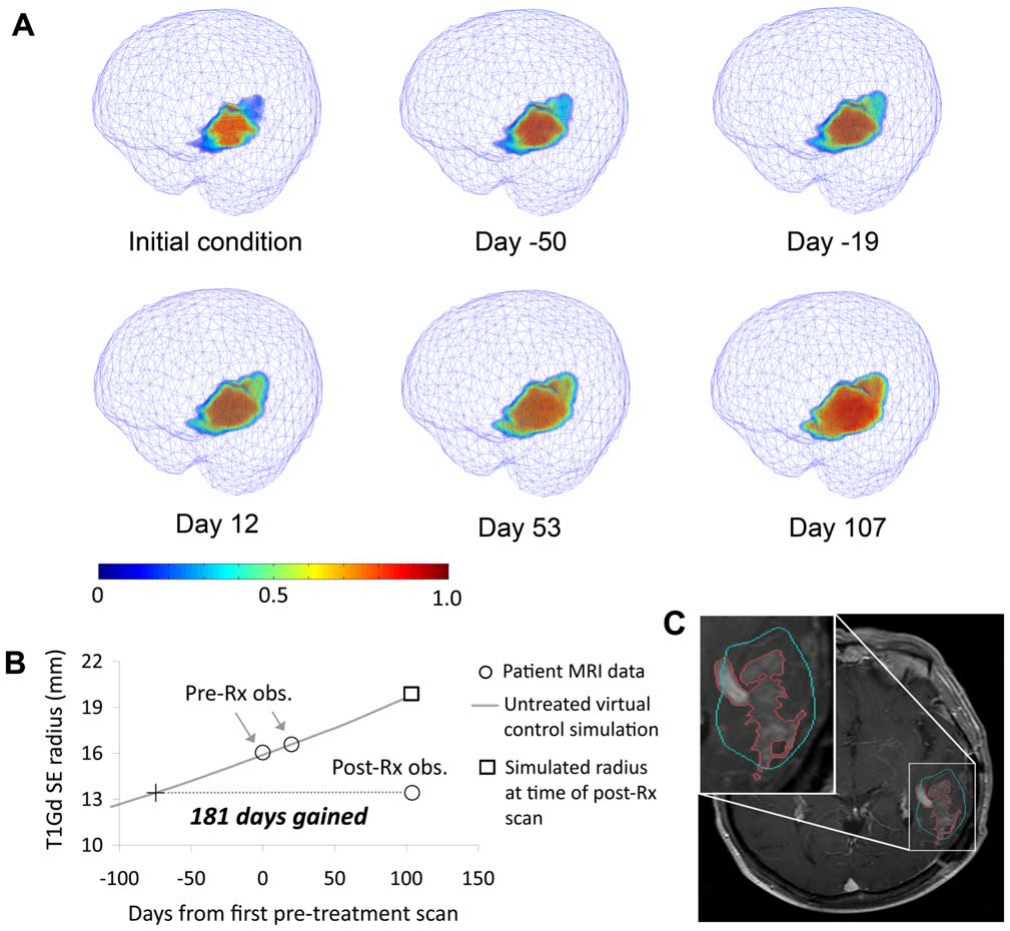
Growth of an untreated virtual control (UVC) tumor for a 57 year-old patient with a left fronto-parietal lobe glioblastoma. *a*: Volumetric images of the untreated virtual control at six time points where Day 0 is the time of the patient's first pre-treatment MRI scan. Pseudocoloring indicates the tumor cell density, normalized to the maximum cell density of the tissue. *b*: T1Gd spherically-equivalent radius time curve from same simulation showing how the Days Gained score is computed. *c*: Post-treatment T1Gd MRI slice showing actual tumor (red outline) and perimeter of the simulated tumor's T1Gd-enhancing region at the same time point (cyan outline). Image oriented according to radiological convention: patient left is on the right.

**Figure 2:**
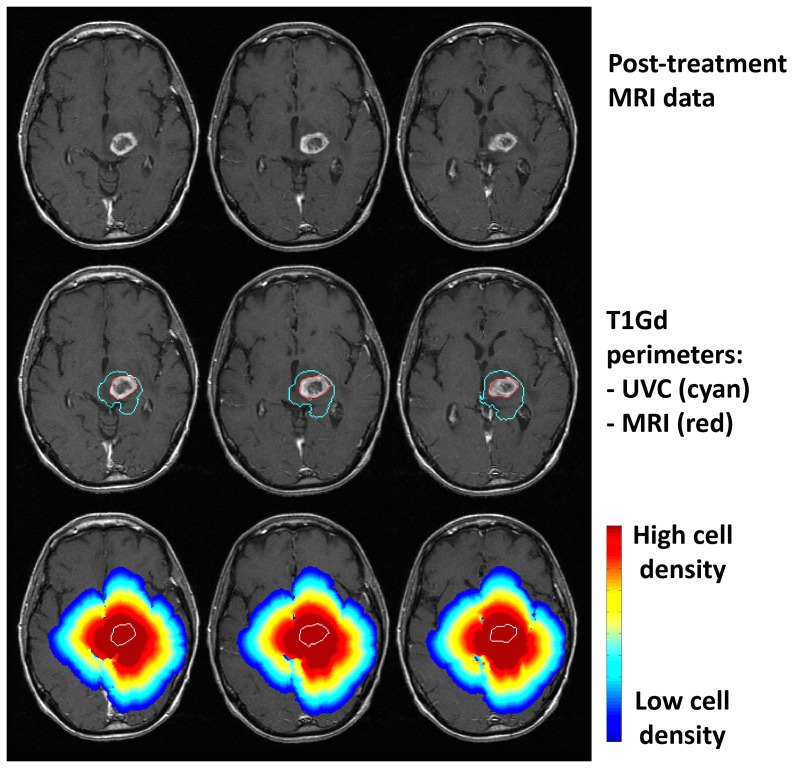
Comparisons between T1Gd MRI data and untreated virtual control (UVC) prediction at post-treatment time point. Patient was 58 years old and underwent biopsy followed by conformal radiation therapy with concurrent temozolomide chemotherapy. Top row: MRI data. Middle row: Actual tumor perimeter (red) with superimposed UVC-predicted tumor perimeter (cyan). Bottom row: full distribution of UVC cell densities showing invasion peripheral to abnormality. Outermost blue cell density profile represents a very low, but non-zero, threshold. Perimeter of actual tumor outlined in white.

Our metric is computed from patient-specific, mathematical models of GBM dynamics that simulate the proliferative and dispersive kinetics of each patient's tumor. These models are based on an established line of research aimed at forecasting tumor dynamics by integrating data from clinical imaging into patient-specific simulations [3], [4], [9], [10], [11], [12], [13], [14]. To generate a simulation from which we can compute a patient's Days Gained score, we first initialize our model using patient-specific data from both the enhancing (visible on T1-weighted gadolinium enhanced MRI – T1Gd) and non-enhancing (visible only on T2-weighted MRI – T2) sections of the tumor [15], [16], [17]. Incorporating both sections, we are able to simulate a continuous distribution of tumor cell densities throughout the brain, including values below the threshold of MRI detection. Thus, we are able to generate a full spatial description of the disease distribution (Figures 1a and 2). To simulate the expansion of the tumor in 3D, we compute the rate of tumor growth from two pre-treatment MRI scans and incorporate this rate into the equations that govern the simulated tumor dynamics [13], [15]. These simulations of tumor growth estimate the glioma cell density throughout the brain at post-treatment time points as if the tumor were untreated (Figure 2). Each of our patient-specific models therefore acts as an *untreated virtual control* (UVC) against which actual, post-treatment tumor data can be compared to estimate a patient's response to a given treatment.

In this study, we explored the ability of our model-based approach to discriminate progression-free and overall survival outcomes among our patients based on MRI scans taken after the completion of first-line, standard-of-care radiation therapy. We applied our Days Gained metric to estimate the amount of time that therapy delayed tumor growth for each patient and found that Days Gained scores separated our study population into two distinct groups with significantly different progression and survival outcomes. Our results indicate that Days Gained scores computed at the time of the first post-radiation scan discriminate time to progression and overall survival, and offer a novel assessment of a patient's response to therapy. They also demonstrate how a personalized response metric can predict clinical outcomes among GBM patients and, more broadly, illustrate the discriminating power of patient-specific computational modeling, an emerging field currently making inroads into various clinical settings [18].

## Materials and Methods

### Ethics statement

All research involving human subjects was approved by the University of Washington and University of California, Los Angeles institutional review boards. Written informed consent was obtained for the collection of all patient data, and the investigation was conducted according to the principles of the Declaration of Helsinki.

### Patients

We collected routine clinical MRIs from 33 newly diagnosed GBM patients at the University of Washington (UW) Medical Center (n = 30) and the University of California Los Angeles (UCLA) Medical Center (n = 3). Inclusion in the study required the existence of two pre-treatment MRIs and at least one MRI taken following radiation therapy. Patients ranged in age from 40–89 years (median 57), with Karnofsky Performance Scores [19] between 60 and 100 (median 90), and Radiation Therapy Oncology Group Recursive Partitioning Analysis classifications of III (n = 5), IV (n = 16), or V (n = 12) [20]. Table 1 presents the clinical characteristics of our patient group. To accurately compute each patient's tumor growth rate, we required a minimum of five days between the patient's two pre-treatment MRI observations [10]. This interval is routine for GBM patients because day-of-presentation (diagnostic) MRI scans and day-of-surgery guidance scans are typically taken several days apart.

**Table 1:** Clinical characteristics of patients.

**Number of patients**	33
**Age in years (median, range)**	57, 40–89
**KPS at diagnosis (median, range)**	90, 60–100
**RTOG RPA classification**	III (n = 5), IV (n = 16), V (n = 12)
**Resection treatment**	
**Biopsy only**	8 (24%)
**Sub-total resection**	9 (27%)
**Gross-total resection**	16 (48%)
**Radiation therapy dose in cGy (median, range)**	6020, 5000–6900
**Patients that received temozolamide concurrent with radiation on Stupp protocol ** [**29**]	21 (64%)
**Patients that received BCNU concurrent with radiation**	4 (12%)
**Days between end of radiotherapy and next MRI scan (mean, range)**	28, 1–72

All patients received radiation treatment and chemotherapy, although exact courses of treatment varied between patients (Table 1). Twenty-five patients (73%) underwent craniotomy with surgical resection of tumor. Nine patients' resections were sub-total, 16 were image-verified gross-total. The remaining eight patients underwent biopsy only.

### Mathematical model

The foundational methods for generating our patient-specific UVC simulations are well-documented [3], [4], [9], [10], [11], [12], [13], [14]. Two patient-specific parameters control the model's dynamics: the tumor's net diffusive capacity (*D*) and the tumor's net proliferation rate (ρ). We compute *D* and ρ using volumetric measures of tumor burden on T1Gd and T2 MRIs at two pre-treatment time points. With these parameters identified, we can simulate the growth of the tumor to the post-treatment time point, accounting for its unique diffusive and proliferative dynamics and the complex architecture of the brain. We have previously shown that these parameters are directly linked to features of tumor aggression including hypoxia visible on [18F]-fluoromisonidazole PET [14], are prognostically significant [4], and can be used to quantitatively predict a patient's response to radiation therapy [10]. The work presented here continues our investigation into the prognostic power of our modeling approach.

The current study incorporates several recent advancements in our modeling work. This is the first investigation we have conducted on a substantial number of patients using anatomically accurate, patient-specific GBM simulations that grow within the constraints of the patient's unique brain geometry. We recently published a study where we simulated patient-specific tumor models within the patient's brain anatomy for two patients [16]; however, our previous studies have either used spherical tumors that grow without spatial constraints [3], [4], [17], or within a canonical, rather than patient-specific, brain geometry [15], [21], [22].

**Figure 3:**
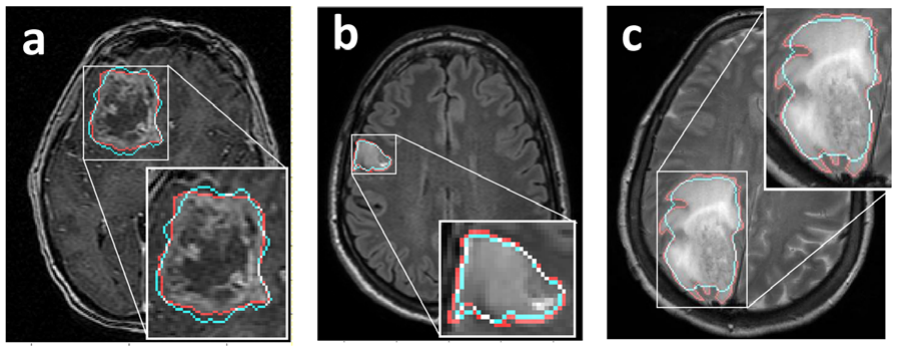
Spatial comparisons between baseline pre-treatment MRI (red outline) and simulation results (cyan outline) seeded with our initial condition. a: large tumor on *T1Gd MRI*. *b*: smaller tumor on FLAIR MRI. *c*: Highly anisotropic tumor growth on T2-weighted MRI. Our simulations produce tight spatial matches to a range of tumors (*a* and *b*), but decrease in accuracy for tumors with high anisotropy (*c*).

Additionally, we developed a novel method for setting the initial cell densities in our simulations so that our UVCs more accurately represent the complex 3D geometry of each patient's tumor. Previously, we started simulations within the brain geometry from a single point source, but this technique requires significant computational time to grow the tumor from a single voxel to its pre-treatment size and does not always reflect the complex 3D geometry of the tumor as seen on pre-treatment imaging. We now begin simulations with a tumor geometry that reflects what is observed on the patient's second pre-treatment T1Gd and T2 MRIs (Figure 3) prior to surgical intervention. This method matches the 3D spatial characteristics of the tumor just prior to therapy and still yields simulation turnaround times comparable to those of a clinical laboratory service.

Specifically, our method for setting the initial tumor cell densities for our simulations begins by reconstructing the T1Gd enhancing volume of the tumor from the patient's second pre-treatment MRI scan. We then erode away the tumor volume by iteratively removing the tumor's perimeter. We continue until the tumor's T1Gd spherically-equivalent (SE) radius has been reduced by 20%. We then iteratively erode the tumor volume as observed on the registered T2-weighted MRI until we reduce its SE radius by the same number of millimeters as the T1Gd radius. Next, we merge the T1Gd and registered T2 scans and set the cell density distribution within the tumor. All voxels within the T1Gd tumor region are set to 80% of the total cell carrying capacity of the tissue. Voxels within the T2 region but outside the T1Gd region are set using a Gaussian-based curve computed based on the voxel's distance from the T1Gd and T2 volume perimeters. We used a similar method to set the densities of voxels outside the T1Gd and T2 region, but in this case the curve was a function of the SE T1Gd and T2 radii and was independent of the voxel's distance from the T1Gd perimeter. For this region we computed the voxel's distance from the T2 perimeter, then set its density using the SE Gaussian curve.

We tested a number of approaches for setting the initial conditions of our simulations and found that the method described here provided the closest spatial match between simulation and data when the eroded tumor (the initial condition) grew to the size observed at the second pre-treatment time point. Figure 3 shows the spatial match between three of our UVC simulations and their corresponding pre-treatment scans. Our method produces tight spatial matches to complex tumor geometry, independent of tumor size and imaging modality. However, Figure 3c shows that, because we iteratively eroded away the perimeter of the tumor when computing our initial conditions, our spatial matches decrease in accuracy with highly anisotropic tumors.

### Image processing

We extracted tumor volumes from T1Gd and T2 patient MRIs using semi-automated segmentation software developed in MATLAB® (R2010b, The MathWorks, Natick, MA) [23]. This software facilitates tumor volume measurement by employing a background subtraction algorithm. The subtraction helps automate the tumor segmentation process because it allows a user to identify the tumor region with a polygon of interest, and an automated edge detection algorithm selects the imaging abnormality within the enclosed area.

We performed all simulations within the geometry of the patient's second pre-treatment T1Gd MRI scan. In creating the initial conditions for the simulation we registered the second pre-treatment T2 scan to the T1Gd scan using the Statistical Parametric Mapping MATLAB toolbox [24].

### The Untreated Virtual Control and the Days Gained metric of response

We applied our UVC simulations to find new response metrics that would offer an assessment of a patient's response to therapy by taking into account the unique spatial and kinetic characteristics of each patient's tumor. For each patient in our study, we computed the growth of their UVC to the time of their first post-radiation scan and then found the time point on the UVC's growth curve where it best matched the actual post-treatment tumor size. The patient's Days Gained score is the amount of time between this time point and the final (post-radiation) time point on the curve (Figure 1b). In cases where therapy reduced the tumor to a size smaller than what was computed on the UVC growth curve, we used a linear interpolation between the values at the first and last simulation iterations to compute the Days Gained score. This is consistent with the radial expansions predicted by the model and already observed in a spectrum of gliomas [11], [25].

Our UVC simulations provide a number of outputs for quantifying patient response to treatment. Each UVC can be used to estimate the tumor volume as it would be observed on T1Gd and T2 imaging and provide an estimate of the total number of tumor cells distributed throughout the brain at any time between the pre- and post-treatment time points. Thus, we have several metrics available for computing a Days Gained score. Out of the metrics we examined, we found that the T1Gd spherically-equivalent (SE) radius discriminated patient survival most significantly [8]. Thus, our focus here is on the predictive value of Days Gained scores based on this output. We compute the T1Gd SE radius from the volume of the simulated tumor that would appear on T1Gd imaging (assumed to be those voxels with cell densities greater than or equal to 80% of the tissue's cellular carrying capacity - 1.0e8 cells/cm^3^) [3], [15]; the SE radius is the radius of this volume, assuming a spherical geometry.

### Statistical analyses

We performed iterative Kaplan-Meier analyses to find optimal Days Gained thresholds that would maximally discriminate our patient group based on PFS and OS. We computed PFS as the interval between the patient's start of cytotoxic therapy and the confirmation of progression using standard criteria as gathered from radiology reports. We computed OS as the interval between the patient's date of diagnosis and their date of death. We censored observations at the time of last follow-up if the outcome in question was not observed. To ensure that our analyses included a substantial number of patients with and without the observed outcome, we limited it to cases where both groups discriminated by the Days Gained threshold contained at least 20% of our total patients. We used the “survival” package for R software (2.10.1, R Foundation for Statistical Computing, Vienna, Austria) to perform the Kaplan-Meier analyses (log-rank test). We considered *p* values less than or equal to 0.05 to be significant.

## Results

### Days Gained scores from untreated virtual control simulations

Figure 1a provides an example of a simulation of one patient's UVC from the pre-treatment initial condition to an estimate of the untreated tumor burden at the time of the first post-treatment scan. The figure illustrates the ability of our modeling approach to simulate a tumor's asymmetric 3D geometry within the confines of the patient's unique brain anatomy. Figure 1b shows the time course of the UVC's simulated T1Gd SE radius from which we computed the patient's Days Gained score and Figure 1c compares the model-predicted, untreated tumor burden to the actual imageable burden as visualized on T1Gd MRI at the first post-treatment time point. Overall, Days Gained scores ranged from −77 to 512 days with a mean of 134 and standard deviation of 111 (negative scores indicate the tumor grew faster than would be predicted by the UVC during the therapy period).

Figure 2 provides a spatial visualization comparing a patient's actual post-treatment MRI to their UVC predictions. To visualize this comparison we isolated the 2D slice within the 3D UVC prediction that corresponds to the imaged MRI slice and superimposed the simulated and actual data. The middle row shows the isocontour for the patient-specific UVC specific to the T1Gd threshold of detection (cyan contour), highlighting the difference between the predicted T1Gd abnormality in the absence of treatment and the actual T1Gd abnormality (red outline) following treatment. The bottom row displays the variation in cell density within the simulated tumor, revealing substantial peripheral invasion.

### Discriminating power of the Days Gained metric

**Figure 4:**
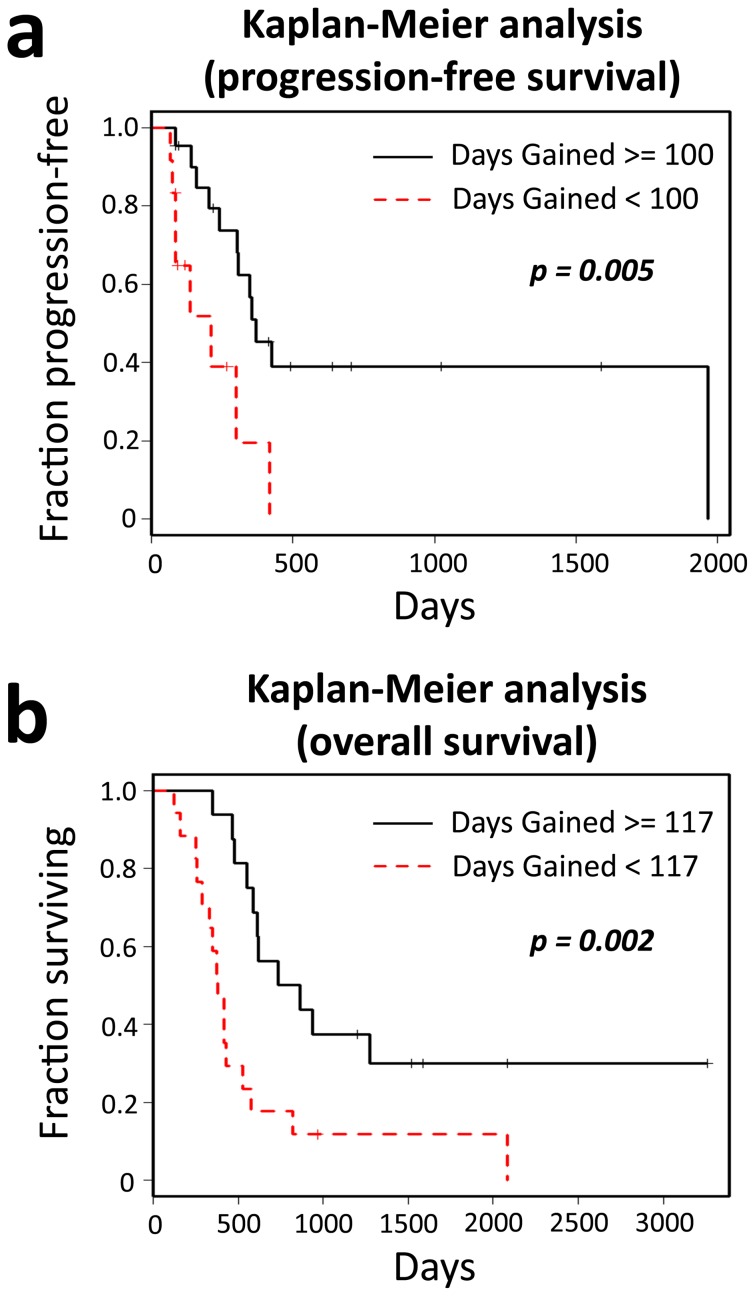
Kaplan-Meier analyses on progression-free and overall survival. *a*: Analysis on progression-free survival data revealed a significant difference between the patients with Days Gained scores greater than or equal to 100 and those with lower scores. *b*: Overall survival analysis also revealed a significant difference between the patients with Days Gained scores greater than or equal to 117 and those with lower scores.

Through our Kaplan-Meier analyses, we found that 100 days is the optimal Days Gained threshold for discriminating PFS and 117 days is the optimal threshold for discriminating OS among our patients. At these thresholds, the Kaplan-Meier analyses based on PFS (Figure 4a) and OS (Figure 4b) both show highly significant differences between patients scoring below the threshold and those scoring at or above it: PFS analysis *p*<0.005, OS analysis *p* = 0.002. As illustrated in Figure S1, there were various Days Gained thresholds that revealed significant differences between PFS and OS outcomes of our patient population. This indicates that our results are robust across a range of Days Gained thresholds and that patients who are more responsive to therapy (those who had larger Days Gained scores) are at an advantage with regard to progression and survival outcomes.

## Discussion

Our results show that Days Gained scores computed using patients' first post-radiation T1Gd scans discriminate PFS and OS among our study population. As a prognostic indicator of PFS and OS, our metric has potential for clinical use during the course of therapy for GBM. It is our hope that Days Gained will eventually provide an early and reliable assessment of a patient's treatment response at the pivotal time point immediately following radiation therapy. Better individual characterization of treatment response will lead to truly personalized therapies and ultimately improve patient outcomes. Because our metric can recognize those patients that are less responsive to their course of treatment, Days Gained may also be useful for early identification of candidate patients for clinical trials. We encourage discussion and debate on the issue of how best to incorporate Days Gained scores into the clinical decision-making process for GBM treatment.

In future studies we plan to test the robustness of our new metric by computing patients' Days Gained scores at multiple post-treatment time points and examining whether our PFS and OS predictions remain prognostic throughout the course of therapy. We also plan to extend our patient cohort beyond the exploratory set investigated here. Additionally, we aim to determine whether our metric can elucidate patient characteristics that are predictive of response to therapy and survival times. For example, we may find that long-surviving patients with low Days Gained scores have certain molecular features that cannot be assessed by imaging alone. With such characteristics identified, clinicians will be better able to use them to tailor therapies on a patient-by-patient basis.

We recognize that several patients in our study showed survival times extending beyond what is typically seen in GBM cases. We therefore examined whether the subset of seven patients surviving longer than three years contained a disproportionate number of individuals with the IDH-1 mutation, a condition that confers a more favorable prognosis for GBM [26]. IDH-1 status, determined by mutation-specific immunohistochemistry, was available for five of these seven patients, none of which were mutants. This suggests that the prognostic value of our response metric, as illustrated through our progression-free and overall survival results, is not due to a biasing presence of IDH-1 mutated patients. Additionally, we found no significant differences in the clinical characteristics listed in Table 1 between the long-surviving patients and the remaining cohort members.

We also recognize that pseudo-progression [27] may have affected our results; many of the post-radiation scans used to compute our Days Gained scores were taken within the post-therapy window where pseudo-progression is likely to be seen. Regardless, our method remained sufficiently robust to discriminate early true-progression and reduced survival following therapy. Pseudo-*response*, a spurious decrease in contrast-enhancing tumor burden that results from anti-angiogenic therapy [7], [28], is another potential confounder for our method as it could falsely raise Days Gained scores. However, none of our patients received anti-angiogenic therapy during the treatment interval we investigated for this study, and it is therefore unlikely that pseudo-response impacted our results. Anti-angiogenics are often applied as treatment following tumor recurrence, and future studies that address the prognostic power of Days Gained during this period will have to account for pseudo-response.

As shown in Figure 3, the spatial matches between our simulations and actual tumor geometry can decrease for tumors with high anisotropy. Although this limitation may be important to consider in subsequent studies that analyze the complex geometry of tumors, we do not anticipate that it significantly affected our results here. In this study we used the tumor's spherically-equivalent radius as the geometric feature for computing Days Gained scores, a metric that minimizes the effects of anisotropy. Had we studied more spatially localized measurements of difference between the model-predicted imageable disease burden and that observed on clinical imaging, anisotropic growth may have more directly affected our results. By applying the spatially simplified metric of spherically-equivalent radius, our results are less affected by differences in anisotropic growth patterns.

We anticipate that our Days Gained metric will have general applicability to GBM patients, and potentially, all patients with contrast-enhancing gliomas. Our study cohort underwent a variety of cancer treatments between their pre-treatment and post-radiation scans, including subtotal resection, gross-total resection, and chemotherapy. Therefore, our metric generalizes to a range of clinical scenarios involving different therapy combinations. Additionally, the metric relies only on data that is routinely collected in the management of glioblastoma; only two pre-treatment and one post-treatment MRI scans are required to compute a Days Gained score. More broadly, although we have focused our study on GBM treatment, our model-based approach may be applicable to a wider range of cancer treatment scenarios where patient-specific tumor kinetics are known, and where diffuse invasion makes it difficult to assess a patient's response to therapy.

Our study illustrates the potential of the emerging field of integrated, patient-specific modeling to impact clinical decision-making and patient outcomes. The next challenge is to translate our computational approach into the clinical setting. Given sufficient manpower for segmenting images, we can currently generate our spatially-resolved virtual controls in the same amount of time required for a clinical laboratory service. We continue to automate our data processing and simulation pipelines, and are currently exploring different software deployment options for bringing our simulation technology into the clinical environment so that providers can make use of our models and our response metric.

## Supporting Information

Figure S1 Color map of p-values from iterative Kaplan-Meier analyses on progression-free survival (PFS) and overall survival (OS). White boxes correspond to statistically significant values. The analyses revealed a range of Days Gained thresholds that separate patients into groups with significantly different PFS and OS outcomes. The most significant p-values for PFS and OS were at the 100 and 117 Days Gained thresholds, respectively.
(TIF)
