# Supplementary figures and images for: Discriminating Survival Outcomes in Patients with Glioblastoma Using a Simulation-Based, Patient-Specific Response Metric
Source: PLoS One. 2013 Jan 23;8(1):e51951. doi: 10.1371/journal.pone.0051951 (PMC3553125; doi:10.1371/journal.pone.0051951)

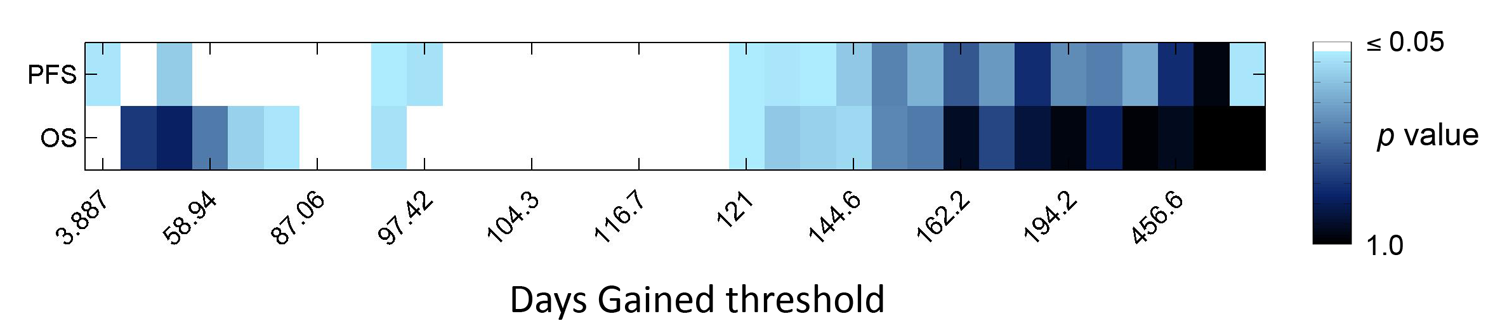

Supplement: Figure S1 — Color map of p-values from iterative Kaplan-Meier analyses on progression-free survival (PFS) and overall survival (OS). White boxes correspond to statistically significant values. The analyses revealed a range of Days Gained thresholds that separate patients into groups with significantly different PFS and OS outcomes. The most significant p-values for PFS and OS were at the 100 and 117 Days Gained thresholds, respectively. (TIF) [file pone.0051951.s001.tif]
